# Supplementary material for: Poria cocus Wolf Extract Ameliorates Hepatic Steatosis through Regulation of Lipid Metabolism, Inhibition of ER Stress, and Activation of Autophagy via AMPK Activation
Source: Int J Mol Sci. 2019 Sep 27;20(19):4801. doi: 10.3390/ijms20194801 (PMC6801774; doi:10.3390/ijms20194801)
Supplement: Supplementary file 1 [file ijms-20-04801-s001.pdf]

Supplementary Materials

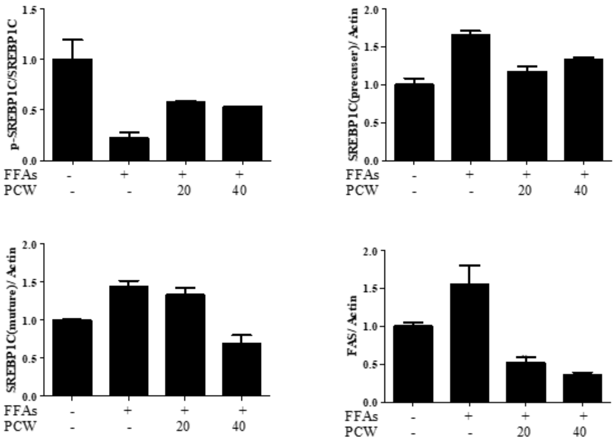

Supplementary Figure S1. PCW inhibited lipogenesis in HepG2 cells treated with FFA. HepG2 cells were treated with FFA (1 mM) and/or PCW (20 or 40 μg/ml) for 24 h. Protein levels of p-SREBP1C, SREBP1C and FAS were analyzed by western blot. Bar graphs represent densitometric analysis of band intensity ratio for p-AMPK/AMPK, p-ACC/ACC.

(A)

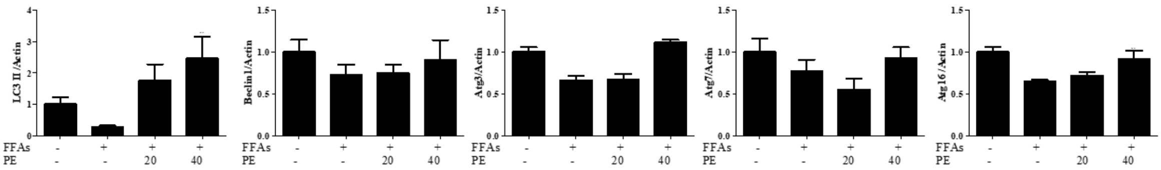

(B)

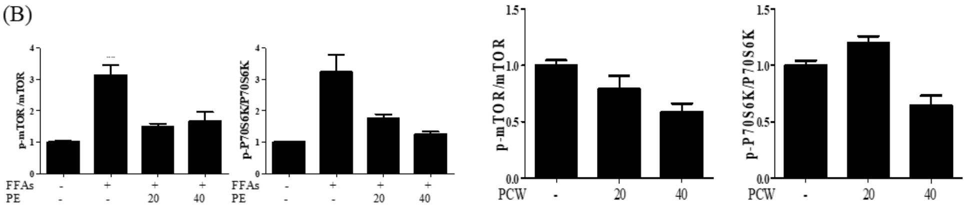

Supplementary Figure S2. PCW activated autophagy in HepG2 cells treated with FFA. HepG2 cells were treated with FFA (1 mM) and/or PCW (20 or 40 μg/ml) for 24 h. (A) Protein levels of autophagy markers were analyzed by western blot. (B) The phosphorylation of p-mTOR/p-P79S6K was determined by western blot. Bar graphs represent densitometric analysis of band intensity.

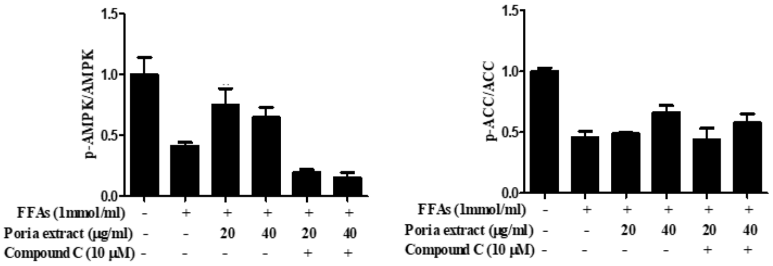

Supplementary Figure S3. Pretreatment with compound C inhibited PCW-mediated activation of AMPK in HepG2 cells treated with FFA. HepG2 Cells were pre-treated with compound C (Comp C, 10 μM) for 3 h and then treated with FFA (1 mM) and PCW (20 or 40 μg/ml) for 24 h. The phosphorylation of AMPK/ACC was determined by western blot. Bar graphs represent densitometric analysis of band intensity ratio for p-AMPK/AMPK and p-ACC/ACC.

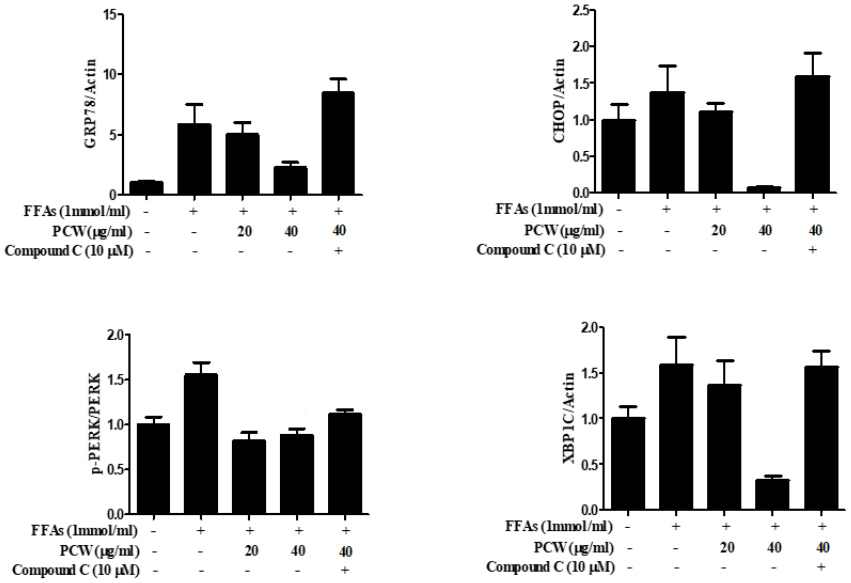

Supplementary Figure S4. Inhibition of AMPK using compound C reversed PCW-mediated induction on ER stress in HepG2 cells treated with FFA. HepG2 Cells were pre-treated with compound C (Comp C, 10 μM) for 3 h and then treated with FFA (1 mM) and PCW (20 or 40 μg/ml) for 24 h. Bar graphs represent densitometric analysis of band intensity.

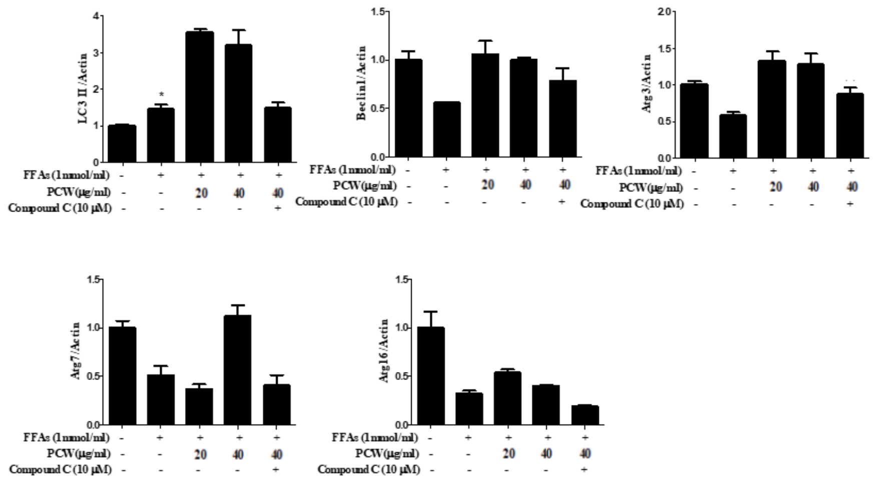

Supplementary Figure S5. Inhibition of AMPK using compound C reversed PCW-mediated reduction of autophagy in HepG2 cells treated with FFA. HepG2 Cells were pre-treated with compound C (Comp C, 10 μM) for 3 h and then treated with FFA (1 mM) and PCW (20 or 40 μg/ml) for 24 h. Bar graphs represent densitometric analysis of band intensity.

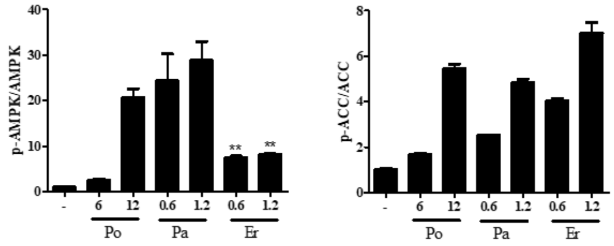

Supplementary Figure S6. **Poricoic acid, pachymic acid and ergosterol activated AMPK in HepG2 cells.** HepG2 cells were treated with Po (6 or 12 μM), Pa (0.6 or 1.25 μM) or Er (0.6 or 1.25 μM) for 24 h. The phosphorylation of AMPK/ACC was determined by western blot. Bar graph represents densitometric analysis of band intensity for p-AMPK/AMPK and p-ACC/ACC.
